# Suitability of Strain Gage Sensors for Integration into Smart Sport Equipment: A Golf Club Example

**DOI:** 10.3390/s17040916

**Published:** 2017-04-21

**Authors:** Anton Umek, Yuan Zhang, Sašo Tomažič, Anton Kos

**Affiliations:** 1 Shandong Provincial Key Laboratory of Network Based Intelligent Computing, University of Jinan, Jinan 250022, China; 2 Faculty of Electrical Engineering, University of Ljubljana, 1000 Ljubljana, Slovenia; anton.umek@fe.uni-lj.si (A.U.); saso.tomazic@fe.uni-lj.si (S.T.); anton.kos@fe.uni-lj.si (A.K.)

**Keywords:** smart sport equipment, sensor selection, strain gage sensor, gyroscope, accelerometer, golf swing analysis

## Abstract

Wearable devices and smart sport equipment are being increasingly used in amateur and professional sports. Smart sport equipment employs various sensors for detecting its state and actions. The correct choice of the most appropriate sensor(s) is of paramount importance for efficient and successful operation of sport equipment. When integrated into the sport equipment, ideal sensors are unobstructive, and do not change the functionality of the equipment. The article focuses on experiments for identification and selection of sensors that are suitable for the integration into a golf club with the final goal of their use in real time biofeedback applications. We tested two orthogonally affixed strain gage (SG) sensors, a 3-axis accelerometer, and a 3-axis gyroscope. The strain gage sensors are calibrated and validated in the laboratory environment by a highly accurate Qualisys Track Manager (QTM) optical tracking system. Field test results show that different types of golf swing and improper movement in early phases of golf swing can be detected with strain gage sensors attached to the shaft of the golf club. Thus they are suitable for biofeedback applications to help golfers to learn repetitive golf swings. It is suggested that the use of strain gage sensors can improve the golf swing technical error detection accuracy and that strain gage sensors alone are enough for basic golf swing analysis. Our final goal is to be able to acquire and analyze as many parameters of a smart golf club in real time during the entire duration of the swing. This would give us the ability to design mobile and cloud biofeedback applications with terminal or concurrent feedback that will enable us to speed-up motor skill learning in golf.

## 1. Introduction

Professional, and often also amateur and recreational sports, are highly competitive. Gaining even a small advantage may result in winning, especially in professional sport. The use of science and technology offers various ways of getting such advantage(s) [1]. One field of research is this area is dealing with the use of smart sport equipment. Examples of smart sport equipment are smart tennis rackets, smart electrical baseball bats, smart golf clubs, and others [2].

Smart sport equipment is one group of smart IoT (Internet of Things) devices that are used in the field of sport and recreation. Smart sport equipment includes at least one sensor and it communicates with another device for sensor data processing and analysis. The communication is wired or wireless, it can be local or through one or more networks [3]. The processing can be performed locally, by a (mobile) device located near the smart sport equipment, or remotely, for example in the cloud.

**Figure 1:**
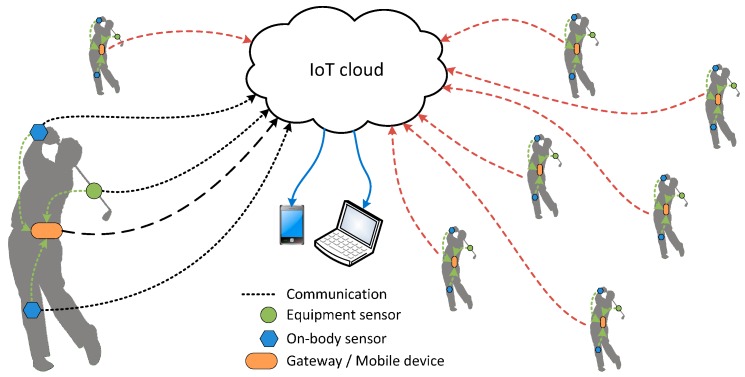
Simplified architecture of IoT sport applications using smart sport equipment and wearables with integrated sensors. Smart sport equipment and wearables send sensor data to the IoT cloud directly or through the gateway. Sensor data processing can be performed locally by the mobile device or in the cloud. Results can be checked by any connected device.

Smart sport equipment connected to the internet forms a group of sport IoT devices. Another group of sport IoT devices are wearable devices attached to the user’s body. Both groups of the abovementioned devices can be used in various mobile and IoT sport applications. A simplified architecture of IoT cloud for sport applications is shown in Figure 1. In general, every IoT sport application implements processes of data acquisition, data transmission, data processing, data storage, and presentation of data and/or processing results. While data acquisition is always at the beginning and data presentation is at the end of the process chain, the rest of the processes are not necessarily always in the same order. On the basis of the above said, three basic groups of IoT sport applications can be identified:

(a) In the classical IoT application sport IoT devices send their data directly to the cloud. In Figure 1 this is shown with black dotted lines. Data is processed and stored in the cloud. The downsides of such application are possible problems with the synchronization of data from independent sport IoT devices, which is caused by different latencies of their independent transmission channels;
(b) To avoid the abovementioned problems, sport IoT devices first send their data to the gateway as shown by green dotted lines in Figure 1. The data is synchronized and processed locally. The results are sent to the cloud for storage and presentation (see black dashed line in Figure 1). The downside of such applications is the possible need for large processing power, which might not be available locally;
(c) The hybrid solution is to collect and synchronize IoT devices data locally. The merged raw data stream is then sent to the cloud for processing, storage and presentation. The raw data path is presented by green dotted lines and black dashed line in Figure 1.

Data acquisition is done by the smart sport equipment sensors and on-body sensors in wearable devices; the presentation of results is performed by connected devices, as shown by blue lines in Figure 1. Many different sport IoT applications belonging to the one of the above basic groups are envisioned to be active at the same time. In Figure 1 they are represented by the minimized silhouettes connected to the cloud by red dashed lines.

The pace of development of smart devices has been increased by recent advances in semiconductor sensor technology, wireless communication, and related technologies [4,5,6,7,8,9,10]. According to [8] the key enablers of Internet of Things are MEMS and sensors. In most cases, smart devices (things) include sensors, microcontrollers, and low power wireless communication. Used together they pave the road to smart system; among others in the fields of health, wellness, recreation, rehabilitation, and sports.

In this paper we focus on the study of smart sport equipment on an example of a smart golf club equipped with several sensors of a different type. The smart golf club is envisioned to be used in various mobile and IoT applications; for example, in a real-time biomechanical biofeedback application aimed to accelerate motor learning of a proper golf swing [11]. The contributions of this paper are:

(a) Validation of SG sensors for golf club bend measuring;
(b) Comparison of several different types of sensors for golf swing performance analysis;
(c) Golf swing technical error detection using only SG sensor signals.

The remainder of the paper is organized as follows: Section 2 is dedicated to motivation, smart sport equipment’s state of the art, and sensors integrated into sport equipment. Section 3 describes the golf club experimental system setup, measurement process, laboratory, and field tests. Results are presented and discussed in Section 4 and Section 5. Section 6 concludes the paper and presents the planned future work.

## 2. State of the Art and Motivation

In the last years the explosion in the number of various devices with one or more sensors has been evident. The trend of measuring and quantifying our activities and our state is on the increase. The main focus of research and development is dedicated to wearable devices used for many different applications and purposes; from simple state detection devices to highly complex expert systems in professional sport [1,2,12,13,14,15,16,17,18].

In many sports the equipment is an inseparable element of the action. Athletes use sport equipment as a tool or medium through which their energy and actions are transferred into a desired result. Measuring and quantifying the actions of the athlete and the response of the sport equipment is expected to prove beneficial to athlete performance improvement. Various sensors can be integrated into smart sport equipment; they should measure the relevant and desired quantities, but they should not interfere with the sport activity itself. Therefore sensors must be lightweight and small-size, they should not alter the properties and functionality of the equipment, and they should not physically obstruct the activity. For the detailed quantification of sport activities a number of physical and physiological quantities should be measured simultaneously; from heart rate and body temperature, to exerted forces, material bends, accelerations, and rotation speeds, among others.

At this time wearable sport devices and equipment predominantly include motion sensors: accelerometers, gyroscopes and sometimes also magnetometers. Such devices are called inertial measurement units (IMUs) [12,13,14,15]. Examples of sport equipment with integrated IMU, such as smart tennis rackets, smart electrical baseball bats, smart golf clubs, or smart balls, are already on the market or are expected to be available in a few years [1,16]. While motion sensors are sufficient to measure movement dynamics and short term positions, they cannot reliably measure other important quantities needed in sports, such as force and bend [15,17]. Important complementary sensors in sport are integrated into sport equipment. The use of the concepts of sensor fusion and smart sensors can be applied to various sports and types of analyses [15].

Our motivation is to study the usability of sensors complementary to IMUs, which can be integrated into sport equipment. Our first goal is to explore the general concepts, properties, and effects connected to the use of complementary sensor. We present this topic in the form of feasibility study of different sensors used for a golf swing analysis. For example, to accurately record the bend of the club shaft at impact, which is one of the important parameters of the golf swing, IMU is not appropriate because: (a) if mounted near the club grip, it is not accurate enough, (b) if mounted near the club head, the acceleration exceed its measuring range and the mechanical stress is too high. An alternative type of sensor should be used to measure the golf club shaft bend [18]. SG sensors have been found appropriate for the job as they measure the strain in the material. In our case the strain is caused by club shaft bending during the swing. SG sensors are small-size and lightweight and fulfill the requirements of a sport equipment sensor.

In our future work we plan to focus on developing real-time biofeedback systems and applications used for accelerated motor learning support in sport and rehabilitation. While many simple and undemanding biofeedback applications need only one sensor type, more complex and more demanding biofeedback applications might need a number of various type sensors to accomplish the given task. For example, some obvious golf swing errors, such as excessive head movement, can be detected only by accelerometer or gyroscope; more specific and subtle golf swing errors might be detectable only by using sensor fusion algorithms on a number of different sensors working in parallel. While this paper focuses on experiments for identification and selection of sensors that are suitable for the integration into a smart golf club, our final goal is the use of those sensors in real time biofeedback applications that will be based on smart sport equipment.

## 3. Experimental System

Analysis of sport activities requires measurements of various physical and physiological quantities. In this paper we focus on the measurement of some of the physical quantities that are related to the functions and operation of the smart sport equipment, such as acceleration, velocity, angular velocity, rotation angle, force, power, energy, material bend, frequency of vibration, and others. Various types of sensors are used for acquiring the above quantities or the quantities that can be used to calculate the desired one. For example, acceleration of the sport equipment can be acquired directly through the accelerometer readings, its speed must be calculated by integrating the acceleration at the known initial speed.

For the selection of the appropriate sensors integrated into sport equipment several factors have to be taken into account. The most important are activity (movement) dynamics and analysis duration. In connection to the aforementioned we can define the appropriate sensor measurement range, sampling frequency, accuracy, and precision. For example, high dynamic movements of the equipment that might collide with or hit some other object require large measurement ranges and high sampling frequency of the sensor. Accuracy and precision requirements depend on the nature of application and also on the duration of analysis.

The selection of sensors for our special case of the smart golf club is bounded by the following conditions: the golf swing is a highly dynamic movement where the accelerations of the golf club parts can reach very high values, far exceeding the available accelerometer measurement ranges that are typically ±16*g*_0_. Similar observations are valid for the gyroscope as well. When the club head hits the ball, or worse, when the club hits the ground, the impact causes high frequency vibrations. Such vibrations can be measured only with sensor devices allowing high sampling frequencies. Bending is very important in golf swing; improper bending indicates improper swing. For this purpose we chose to use the SG sensors that measure golf club shaft strain that is directly dependent on the shaft’s bending. The strain is a relative change of the dimensions of the material under load. It is defined as Δ*x/x*, where *x* is a dimension in [m]. The unit for strain is dimensionless and is commonly presented as *ε*. To satisfy the above requirements we have chosen to put the accelerometer and gyroscope sensor device just below the club grip and two orthogonally placed SG sensors in the upper section of the golf club shaft. The details are given in the following subsections.

### 3.1. System Setup

The smart golf club measuring system presented in this paper includes: (a) two single grid strain gage sensors SGD-3/350-LY11 from Omega (Norwalk, CT, USA), which measure the golf club shaft bend, (b) 3-axis MEMS accelerometer and 3-axis MEMS gyroscope, which measure acceleration and angular speed of the golf club. The latter two sensors are a part of the independent Shimmer 3 IMU (Shimmer, Dublin, Ireland) equipped with Bluetooth communication interface. Strain gage sensor signals are acquired by the cRIO professional measurement system with module 9237 (National Instruments Corporation, Austin, TX, USA). The measurement results gained by the SG, accelerometer, and gyroscope sensors are monitored and validated by the Qualisys™ professional high-precision optical tracking system (Qualisys Inc., Göteborg, Sweden) [19].

We conducted our experiments in several phases: sensor mounting and performance testing, sensor calibration, sensor validation, static tests, field tests. All experiments, except the last one, were conducted in the laboratory environment. The experimental system elements are described in the following subsections.

#### 3.1.1. Sensors

**Figure 2:**
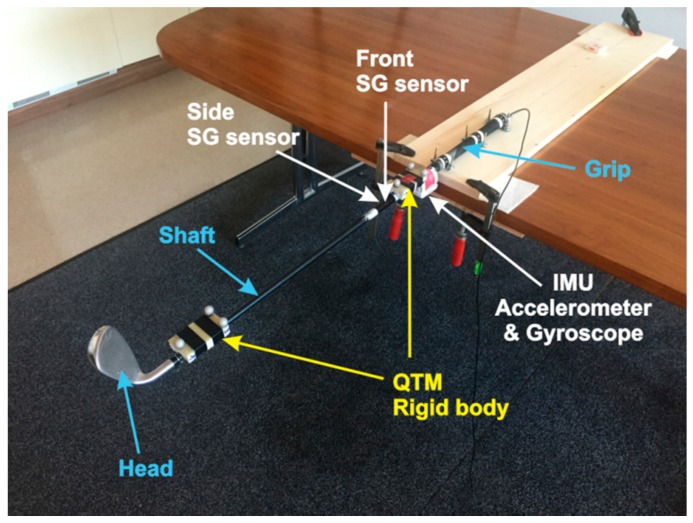
Smart golf club equipped with various sensors fixed to the shaft at different positions: the IMU device with 3-axis accelerometer and 3-axis gyroscope is just below the grip; two SG sensors pasted orthogonally to each other along the shaft’s axis; Two independent rigid bodies used by the QTM optical system are fixed at the top and at the bottom of the shaft.

For measuring the acceleration and rotation of the golf club we used a Shimmer 3 IMU with integrated 3-axis accelerometer and 3-axis gyroscope. The Shimmer 3 device allows sampling frequencies of up to 2048 Hz. According to our outdoor field test experience, the Shimmer 3 device can reliably stream sensor data up to sampling frequencies of 512 Hz, which was used in our experiments. The accelerometer’s dynamic range is up to ±16*g*_0_ and the gyroscopes’ dynamic range is up to ±2000 deg/s. The precision of both is 16 bits per sample. In the experiments the Shimmer 3 device is fixed to the club’s shaft just below the grip, as seen in Figure 2.

**Figure 3:**
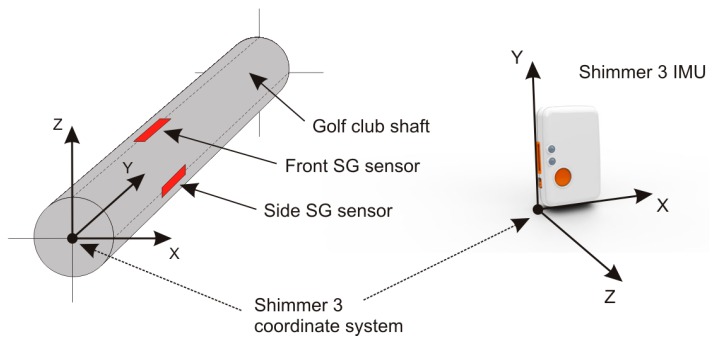
Graphical representation of the smart golf club shaft with two orthogonally placed strain gage sensors measuring the bends of the shaft in two orthogonal directions. The front SG senor is placed in the direction of the Z axis and side SG sensor in the direction of the X axis of the Shimmer 3 IMU device; both measured from the axis of the shaft.

Measuring of the golf club bend is performed by two SG sensors that are pasted parallel to the shaft’s axis and orthogonally to each other as seen in Figure 3. They are placed a few centimeters below the Shimmer 3 IMU. The front SG sensor measures the bend in the direction from and to the golf player (toe up–toe down); the side SG sensor measures the bend in the left-right (lead-lag) direction. The graphical representation of their orthogonal placement is shown in Figure 3 and their exact placement on the golf club shaft in Figure 2.

#### 3.1.2. Optical Reference System

**Figure 4:**
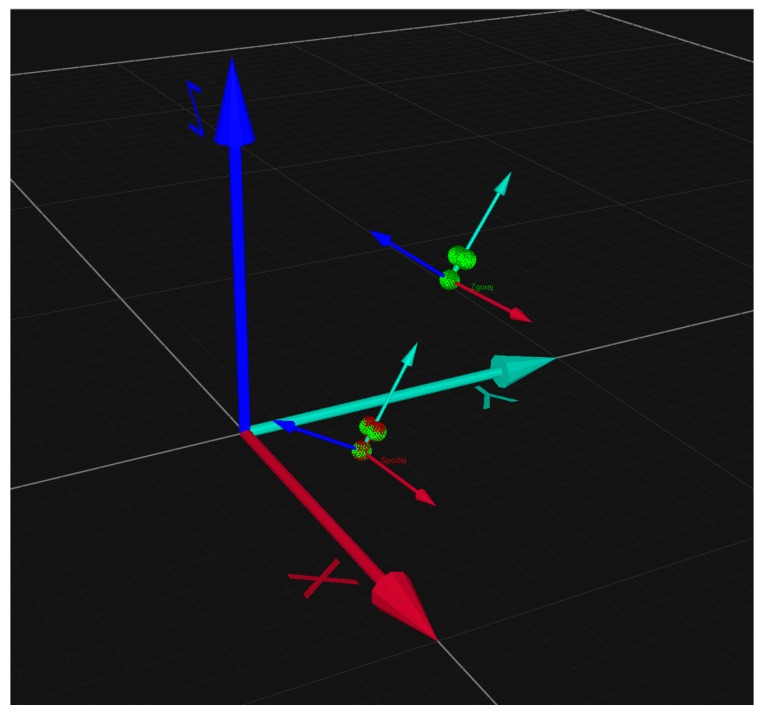
3D rigid body tracking with Qualisys™ Track Manager. The positions of two independent rigid bodies attached to the golf club shaft at one point in time are shown in the global coordinate system XYZ. Each rigid body is composed of three markers and has an independent local coordinate system with the origin in one of the markers.

We used the optical motion capture system Qualisys™ as a reference for the 3D rigid body tracking. Qualisys™ is a professional, high-accuracy tracking system [19] with eight Oqus 3+ high-speed cameras that offers real-time tracking of multiple predefined rigid bodies. The placement of the two independent rigid bodies, each with three reflective markers, can be seen in Figure 2. One rigid body is placed just below the IMU and the other just above the head of the golf club. The position of both rigid bodies in 3D space is shown in Figure 4. The global coordinate system of the test space and two independent local coordinate systems of rigid bodies can also be seen in Figure 4.

The exact position and orientation of the rigid body is captured by the Qualisys™ Track Manager (QTM) software application that defines the global coordinate system, determines the absolute position of each tracked marker, and calculates the orientation of rigid bodies. Relative rotations between bodies can be calculated as well. The marker capture frequency of the QTM can be as high as 1000 Hz, its real time streaming operation, needed in our system, is limited to 50 Hz [19].

#### 3.1.3. Sensor Signal Acquisition

Sensor signals are synchronized and processed by the distributed LabVIEW™ application running simultaneously on the laptop and cRIO platform. Laptop and cRIO are interconnected by Wi-Fi connection. The sampling frequency of the main LabVIEW module running on the cRIO is 500 Hz. The system receives three independent data streams:

(a) QTM application streams the rigid body orientation over the Ethernet connection to the LabVIEW module running on the laptop with the frequency of 50 Hz;
(b) Shimmer 3 IMU streams the accelerometer and gyroscope sensor signals over the Bluetooth connection to the LabVIEW module running on the laptop with the frequency of 512 Hz;
(c) SG sensors are connected by wire to the SG module NI 9237 inserted into the cRIO platform. The sampling frequency for SG signals is 500 Hz.

#### 3.1.4. Sensor Signal Processing, Labelling, and Storage

**Figure 5:**
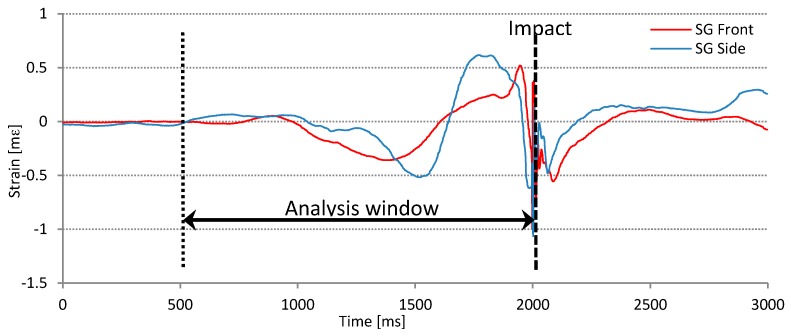
Recording and analysis window of a single swing. Swing recording time is 3 s with the impact point set at 2 s. The analysis window has the width of 1.5 s.

As described in Section 3.1.3, acquired sensor signals are synchronized and processed by the LabVIEW™ application. Because the communication paths from IMU and SG sensors have different latency, we perform impact detection separately for the SG signals and IMU signals. After real-time streamed sensor signals are aligned by their impact samples, they are segmented into separate swings. Each swing contains 1500 samples, where impact sample is always at index 1000. At the defined sampling frequency of 500 Hz the duration of each recorded swing is 3 s and impact point is at 2 s. The relations between the recording and analysis window are presented in Figure 5. In the graphs presented later in this paper we plot only swing signal samples between indexes 250 and 1000 (impact) or 1.5 s time frame. For our analysis, aimed at later use in real time biofeedback system, this is the most significant part of the swing. The signal recorded before the start of the movement is considered to be noise and signal after the impact is irrelevant for real time biofeedback as it occurs too late for action. Even more, for motor learning accelerating application the feedback should happen before the downswing phase, what is approximately 250 ms before impact.

Synchronized and segmented raw sensor signals are saved to the database. In addition to raw data, with each swing we also save a number of parameters defining the conditions and settings of the experiment at the time of the swing. Each swing is labeled with swing outcomes such as the distance, swing shape, player’s evaluation, and similar. The application also allows reviewing and replaying the swing data from the database.

### 3.2. Experiments

#### 3.2.1. Laboratory Tests

Laboratory tests are performed primarily to study the properties and the performance of SG sensors used for our smart golf club. The final goal of the laboratory test is the calibration of SG sensor readings and validation of their use for measuring the golf club bend.

**Figure 6:**
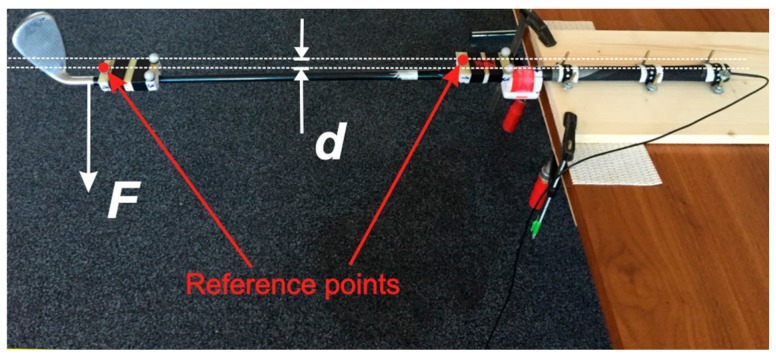
Bending test for SG sensor calibration and validation. The golf club shaft bends under the force exerted to the bottom of the shaft. Bending is calculated from the height difference of two markers measured by the QTM system.

A static test is performed with the club’s grip firmly affixed to a massive table as shown in Figure 2 and Figure 6. The shaft bends for the deviation *d* under the known mass *m* that exerts the force *F* = *mg*_0_ to the shaft just above the club’s head. The bending deviation *d* is measured directly by the high precision reference QTM system through the positions of the two reference points of the two independent rigid bodies, as shown in Figure 6. The shaft’s bending strain in the up-down (toe up-toe down) and left-right (lead-lag) actions are measured by the two orthogonally placed strain gage sensors as shown in Figure 2 and Figure 3. The bend in each direction is then calculated from the corresponding strain.

**Figure 7:**
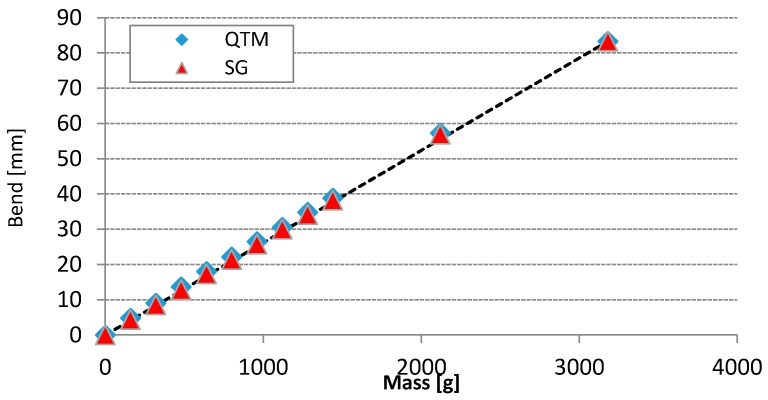
Front SG sensor calibration and validation. The bend is measured directly by the QTM system and indirectly by the SG sensor. The horizontal axis shows the mass of the applied weight in [g], the vertical axis shows the bend of the club head in [mm].

The graph in Figure 7 shows the bend of the golf club head in [mm] under application of a known mass exerting the known force *F* = *mg*_0_. The bend is measured by the QTM system and is practically linear to the applied mass in the test range. The SG sensor readings are multiplied by the constant calibration factor to calculate the bend in [mm]. The constant calibration factor is determined from the slopes of the QTM and SG graphs. As seen from the graph in Figure 7 the bend calculated from the SG readings is also practically linear to the applied mass, thus we conclude that SG sensors are validated for measuring the bend of the golf club. For obvious reasons, an IMU device was not used in static tests.

#### 3.2.2. Field Test

After the calibration and the validation of SG sensors in the static laboratory tests described in Section 3.2, we performed the pilot set of dynamic field tests to prove the correct operation of the experimental system elements and to acquire the first set of measurements for the first analysis.

**Figure 8:**
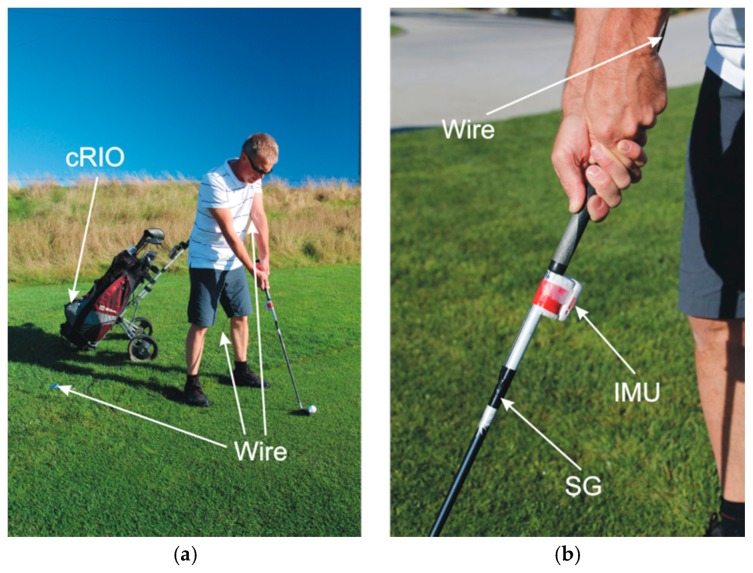
Field test. The movement of the golf club during a typical golf swing is recorded by the SG sensors and the IMU device. (**a**) SG sensors are connected by wire to the nearby cRIO device. (**b**) The IMU device is connected wirelessly to the nearby laptop (not shown in pictures).

Dynamic field tests include two SG sensors and an IMU device. The SGs are connected to the cRIO device, IMU is connected to the laptop. cRIO and laptop are running the distributed LabVIEW application. As seen from Figure 8a, the SG sensors are connected to cRIO by a thin wire. The IMU device is connected over a Bluetooth connection to the nearby laptop (not shown in Figure 8). The laptop is used for communication with the IMU for monitoring and controlling the measurement process. cRIO is running on batteries and is placed into the golf trolley. The exact placement of SG sensors and IMU device is seen in Figure 8b.

The dynamic test is performed by several experienced amateur golf players and two professional golf players. They consecutively perform a number of golf swings. In addition to that the professional golf players are asked to perform a number of swings with a known and controlled technical error. All players are using the golf club shown in Figure 2.

## 4. Results

Field test results include signals from SG and IMU sensors, while the QTM optical tracking system was not used for its impracticability. The use of inertial sensors is not new to golf. There are many applications, such as [20,21,22,23,24,25,26,27], that use accelerometers, gyroscopes, or both, for golf swing tracking analysis. One example of the use of optical tracking system for golf is presented in [28], where the authors use a professional Qualisys optical system for detection of technical errors in putting practice based on the deviation between the reference motion and the motion under analysis. Such optical systems are unfortunately very expensive and highly impractical for the field use. Our primary focus was with SG sensors, which are not commonly used for this purpose.

The most often use of IMU devices in golf is body and/or club motion tracking. The authors of [20,21,23] use accelerometers and gyroscopes attached at the grip end of the club for its position and orientation tracking. In [22] an IMU device attached to the player’s hand for the advice on the selection of the most appropriate club is used. An example of an IMU device attached to the putter head is presented in [26]. The most limiting factor in all the above papers is the dynamic range of accelerometers and gyroscopes (see Section 3.1.1) in regard to the suitable place of attachment: (a) if sensors are attached close to the club head, the motion dynamics (except with putter) will exceed their measurement range; (b) if sensors are attached close to the club grip they cannot measure the club shaft bend. Our system complements the IMU devices with SG sensors. It is capable of measuring the club shaft bend with SG sensors and the motion of the club with the IMU device. The primary goal of our research is not golf swing tracking, but rather detection of any deviation between the reference swing and the swing under analysis.

SG sensors have been used before in golf, as presented in [18,29,30]. Both studies were oriented primarily to the understanding of golf club performance and not to the analysis of the golf swing execution as it is the case in our paper. Our research studies SG sensors, accelerometers, and gyroscopes and their suitability for golf swing technical error detection. SG sensors are very suitable for integration into the golf club because: (a) they have no practical limitation in their dynamic range; (b) they can measure the forces even if the club is in standstill position; (c) they measure the club shaft bend, what is extremely difficult with IMU devices.

### 4.1. Repeatability

**Figure 9:**
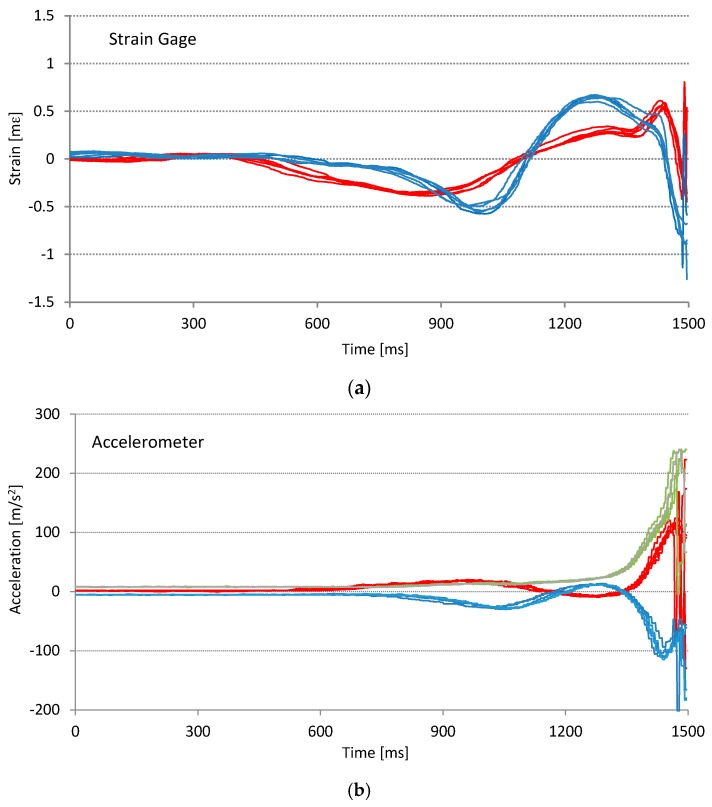

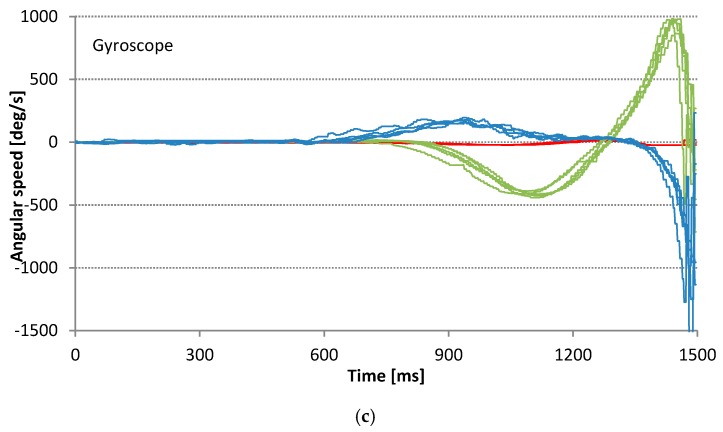
Measurement repeatability test. Graphs show sensor signals for the same series of five consecutive swings performed by a professional golf player: (**a**) two orthogonal SG sensors; (**b**) 3-axis accelerometer; (**c**) 3-axis gyroscope. In graph (**a**) blue plots represent the front sensor and red plots represent the side sensor, while graphs (**b**,**c**) red, green, and blue lines represent X, Y, and Z axis respectively.

In the field test we performed several sets of swings. The first set was aimed to test the repeatability of sensor readings. For this purpose we have asked a professional golf player to carefully perform a series of consistent golf swings. We measured the response of two SG sensors, 3-axis accelerometer and 3-axis gyroscope. For the repeatability test a series of five best consecutive swings is used. Sensor readings from the start of the swing to the impact are shown in Figure 9.

**Figure 10:**
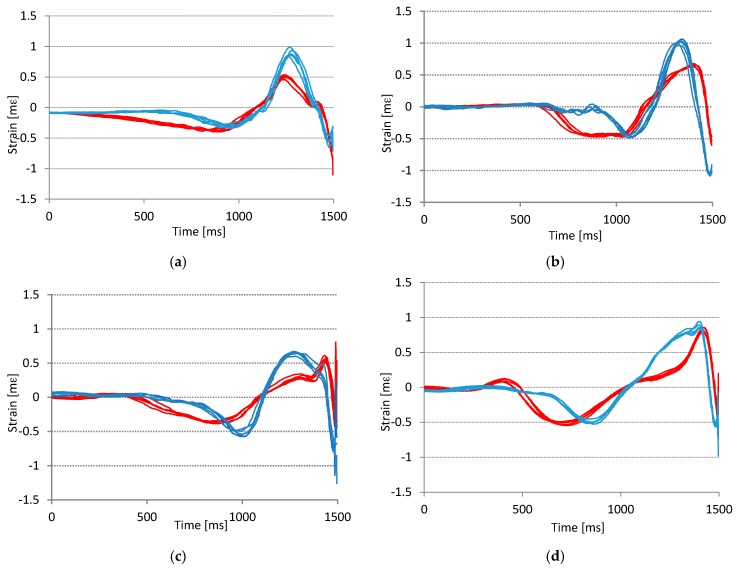
Player’s signatures of five successful consecutive swings of four different golf players: graphs (**a**,**b**) show swings of two experienced amateur players; graphs (**c**,**d**) show swings of two professional golf players. All graphs show the response of two orthogonally placed SG sensors, where blue plots represent the front sensor and red plots represent the side sensor. Signals show that each player has a distinctive sensor response (signature).

The results in Figure 9 and Figure 10 show the variations in sensor signals that are caused by variations of swing performance. Sensor imprecision due to various factors (noise, bias drift, scaling factor drift, etc.) is negligibly small in comparison with effects of swing performance variations caused by the inconsistency of the player. From the graphs in Figure 9 and Figure 10 it can be concluded that all sensor signals during the series of consistent swings show high repeatability in both time and amplitude. It is expected that different features can be extracted from different sensors. While SG sensors measure the force exerted to the golf club and are closely correlated to the player’s action, IMU sensors better reflect the golf club response.

### 4.2. Strain Gage Sensors

One of the aims of this paper is to examine the usability and suitability of SG sensors for golf swing analysis. Figure 10 shows the readings of the front and side SG sensor of four different golf players. Each one of them performed five consecutive swings.

From the graphs in Figure 10 we can conclude, that considering the findings about the repeatability from Section 4.1, all players performed consistent swings. The swing consistency is also evident from the experiment records metadata where all the players subjectively labeled the swings as correctly executed.

Apart from the repeatability and consistency, the graphs in Figure 10 show that all tested players have their characteristic and distinctive signal shapes—player’s signature. For example, the player in the graph in Figure 10b starts the swing at approximately 600 ms, while other players start the swing at approximately 200 ms and 400 ms. Similarly we can observe that players in graphs Figure 10a,c have a bounce in the red signal just before the impact, while the other two do not. Other similar examples can be derived from Figure 10.

Even the top professional golf players have different golf swings. Their golf swings depend on their body constitution, their physical abilities, and their personal style. The objective of golfers in learning or training is not to repeat the swing of another player, but to learn to repeat their own best swing; for example, the swing that results in the intended ball flight. Each golfer should strive to be able to repeat the good swing over and over again.

**Figure 11:**
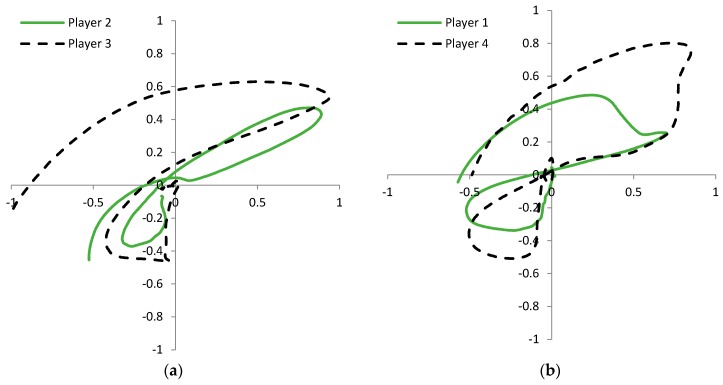
Player’s signatures shown in 2D strain plot indicate that each player has a very distinctive sensor response. Graph (**a**) shows signatures of experienced amateur players; graph (**b**) shows signatures of two two professional golf players. The strain in both axes is given in [*mε*]. The horizontal axis shows the strain of the front sensor and the vertical axis shows the strain of the side sensor.

A more intuitive representation of the difference in players’ signatures is given in Figure 11. The 2D strain graph is created by plotting the readings of the side SG sensor to the horizontal axis and the readings of the front SG sensor to the vertical axis of the graphs. Figure 11a shows the signatures of two amateur golf players and Figure 11b the signatures of two professional golf players. It is obvious that the shape of the plots does not depend on the quality of the player. Based on the observed plots, we expect that the identification of a player is possible with a high probability. The player identification is not one of the main focuses of this research; however the results in Figure 10 and Figure 11 can give us important directions for the system design. It is important to consider the fact that correctly performed swings of different users are different. The consequence of this observation is the need for personalization of the swing error detection application; the application learning phase will have to be performed separately for each player.

### 4.3. Detection of Golf Swing Technical Errors

One of the final goals of every golf swing analysis is the detection of technical errors made during the swing execution. Through the identification and elimination of error causes players can improve their swings and consequently advance in mastering golf.

Traditionally, error analysis is done after the golf swing, in post-processing, giving players a terminal feedback. One of our goals is to develop and implement an application that gives players (bio) feedback in real time. That means that a player is notified about possible technical error in swing execution as soon as the error can be detected; if possible, before the downswing phase.

**Figure 12:**
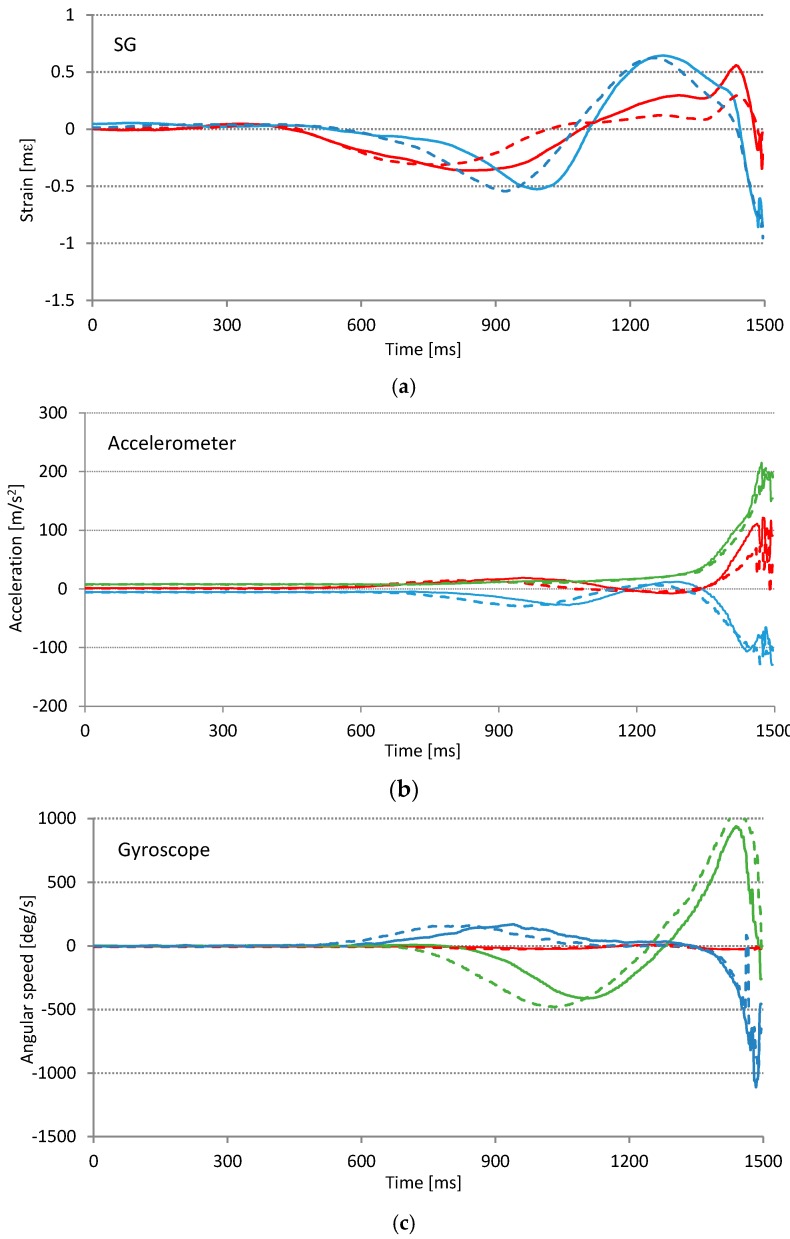
Technical error detection. Averages of five consecutive swings: (**a**) two orthogonal strain gage sensors; (**b**) 3-axis accelerometer; (**c**) 3-axis gyroscope. Solid lines represent averages of five consecutive successful swings; dashed lines represent averages of five swings with “slice” technical error. Traces of the same color are distinctively different from each other. We expect that technical error detection is possible. In graph (**a**) blue plots represent the front sensor and red plots represent the side sensor, while graphs (**b**,**c**) red, green, and blue lines represent X, Y, and Z axis respectively.

All graphs in Figure 12 show that the slice swing can be distinguished from the correct swing. With the accelerometer we observe a slight deviation of the timing in the X and Y axis around the 1000 ms mark, and the amplitude in the X axis just before the impact. Similarly, the gyroscope shows timing deviations in Y and Z axis, while amplitudes are practically the same for both series of swings. The greatest difference in signal traces is observed with SG sensors. Especially important is the amplitude difference at approximately 1250 ms mark that represents the top of the backswing. We find out, that from all of the sensors used in our experiment, SGs are probably the most suitable for the detection of (slice) technical errors.

Another observation is that when comparing all the graphs in Figure 12, the SG sensors are the first that show any response (signals different from zero value) to the performed movement. The reason for this is that they represent the force applied to the golf club, while accelerometers and gyroscopes represent the response (the movement of the golf club). This observation supports the claim that SG sensors, being closer to the cause of the error, can be more suitable for technical error detection. Another advantage, especially for the real-time biofeedback, is that SG sensors show the error earlier in time and therefore allow enough time for possible swing interruption [11].

**Figure 13:**
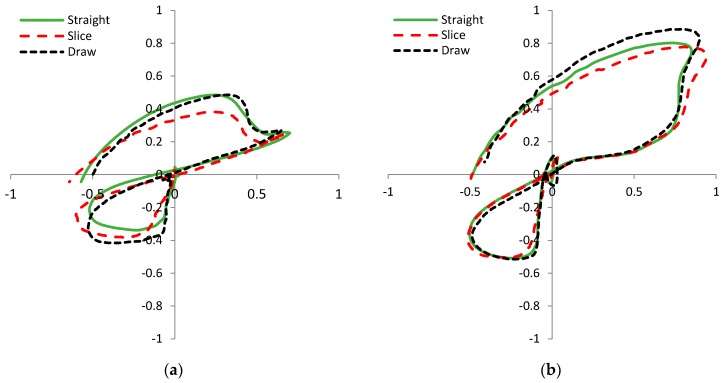
Comparison of 2D strain [*mε*] plot for regular swings and swings with technical errors of two professional golf players: (**a**) Player 1; (**b**) Player 4. Regular swings (straight) differ from swings with technical errors (slice, draw) in one or more swing phases. We expect the identification of technical errors is possible. The strain in both axes is given in [*mε*]. The horizontal axis shows the strain of the front sensor and the vertical axis shows the strain of the side sensor.

To explore this notion further, we plot three different swing shapes: straight, slice, and draw. 2D strain graphs for two professional golf players are given in Figure 13. As expected, shapes of the same swing type differ greatly between the players. This is consistent with the observations from Figure 11, where each player has very distinctive swing signature. From both graphs in Figure 13 it can be observed that trajectories depend on swing shape, but the difference between them is not as great as the difference between players’ signatures in Figure 11. Based on the plots in Figure 13, we expect that the identification of different technical errors, within the swings of the same player, is possible.

### 4.4. Technical Error Detection Results

Detection of swing type in this section is based on signal correlation coefficient comparison. Swing data from *K* swings is grouped by player and swing type *G*(*Player, SwingType*). Metadata *Player* and *SwingType* is a priori knowledge, available for each swing. Each group of swings is characterized by a normalized average sensor signals $x_{ref}^{G}$[*n*], which serve as a reference for comparison with all analyzed swings *x_k_*[*n*], where k∈ {1,…,*K*}. Each signal *x_k_*[*n*] is compared to all group reference signals by calculating the Pearson correlation coefficients *C*(*k,G*) = ρ(*x_k_*[*n*], $x_{ref}^{G}$[*n*]). Detection of the most probable swing type is made according to the max-correlation coefficient criteria. The same procedure is used for all sensor signals.

The results of the technical error detection of the first field tests with a professional golf player are listed in Table 1. Probability of a successful golf swing technical error detection based on signals of different sensors is approximated with a relative frequency of an event. The total number of swings performed by Player 1 is *K* = 67. The detection accuracy achieved by SG sensors is at least as good as the accuracy achieved by the accelerometer or the gyroscope.

Swing error detection accuracy can vary by players. The test results for a mixed group tests with two players and three golf swing errors are presented in Table 3, while experimental settings parameters are listed in Table 2. The number of analyzed swings is K = 84 that include only three of the nine swing types: straight, slice, and draw. The detection accuracy is calculated by taking into account all signal samples from the takeaway (golf swing start) to the impact, when the club hits the ball (*N_imp_* = 750). It is known [11] that the concurrent feedback in golf swing training should be offered before the downswing phase begins. That is approximately at the 625-th sample (*N_FB_*). For this reason it would be very useful to detect a golf swing technical error during the backswing, that is, in the first 625 samples. Results in the last column of Table 3 show that early detection accuracy is lower than the detection accuracy with signal samples up to the impact. The ranking of the results in Table 3 can be compared by grading the 2D SG plots similarity in Figure 13. For both players it is possible to claim that the slice swing error in Figure 13 differs the most and that the measured detection accuracy for a slice swing error is expected to be higher than for other swing errors.

## 5. Discussion

Our primary focus was to assess the usability of SG sensors for golf swing analysis and compare them to more widely used inertial sensors. The tests results are a collection of 213 swings performed by four different players; two professionals and two experienced amateurs.

For the intended application of biomechanical feedback, the exact orientation, velocity and position of the club head are not of great importance. The most important is the “signature” of the proper swing, i.e., the swing that the golfer would like to repeat again and again. The idea is to detect any deviation from this “signature” and “warn” the golfer to suspend the swing as in its early phase, as soon as the error is detected, e.g., already at the beginning of the backswing, so that he does not “learn” improper motion. Experiments showed that improper motion or even improper setup could be easily detected from the bending of the club shaft, easier than from data from inertial sensors placed on the hand of the golfer or at the upper part of the shaft.

We can observe that signal shapes of the same player performing the same swing are practically identical, what is shown in Figure 9 and Figure 10. This confirms golf player consistency and measurement system precision. On the other hand the signal shapes of different players are very dissimilar. Player identification can be acquired from any swing sensor signal, see Figure 10 and Figure 11. Different swing types (errors) from the same player show relatively small difference between themselves (see Figure 13), but probably enough to detect and identify various golf swing technical errors. The first encouraging results gained by a basic cross-correlation signal analysis, and presented in Table 1 and Table 3, confirm our presumption and show that the detection accuracy achieved by SG sensors is at least as good as the accuracy achieved by the accelerometer or the gyroscope. We expect to get more reliable and more detailed results by using more sophisticated data analysis methods on a larger data set, such as presented in [31,32,33,34].

## 6. Conclusions

Smart sport equipment is entering professional and amateur sports at a fast pace. While many tasks can be carried out by using single sensor signals and processing, more complex tasks, such as golf swing analysis, may require more sensors of different types and complex processing.

We have tested the usability of less common SG sensors against the widely used and studied inertial sensors. We found out that for the analysis of the golf swing they complement each other. A sensor fusion scenario is suitable as each of the sensors show some of the important parameters of the golf swing that cannot be acquired by other sensors. SG sensors are especially suitable because bending of the club shaft reveals some information of the swing performance which could not be obtained from inertial sensors, which, due to range limitations, cannot be placed on club head. SGs are thus on the one hand complementary to inertial sensors and in another hand redundant, revealing the same information as an IMU. This redundant part could also be used in combination with inertial sensors in sensor fusion. We expect that for some relatively undemanding applications SG sensors alone are enough. Our future work includes testing of the large group of players with various technical errors and use of more sophisticated data analysis methods.

Our final goal is to develop the system capable of immediate detection of errors and giving appropriate real time (bio) feedback to the player. This includes the development of the system for real time swing analysis and the design of mobile and cloud applications with terminal or concurrent feedback. Such applications will enable golf players to speed-up motor skill learning in golf.

## Figures and Tables

**Table 1:** Probability of swing error detection based on signals of different sensors.

Swing Type	Detection Accuracy		
	Strain Gage	Accelerometer	Gyroscope
straight	0.93	0.69	0.72
slice	1.00	1.00	1.00
draw	0.83	0.67	0.83

**Table 2:** Experimental setting parameters.

Parameter	Notation	Value
Analyzed swings	K	84
Signal samples for error detection	Nimp	750
Signal samples for concurrent feedback	NFB	625
Players		2
Different swing types		3

**Table 3:** Probability of swing error detection from strain gage signals.

Player ID	Swing Type	Detection Accuracy	
		750 Samples	625 Samples
1	straight	0.93	0.88
1	slice	1.00	1.00
1	draw	0.83	0.83
2	straight	0.91	0.73
2	slice	1.00	0.50
2	draw	0.75	0.75
